# Circadian dysfunction and Alzheimer's disease – An updated review

**DOI:** 10.1002/agm2.12221

**Published:** 2022-08-15

**Authors:** Faizan Ahmad, Punya Sachdeva, Jasmine Sarkar, Raafiah Izhaar

**Affiliations:** ^1^ Department of Medical Elementology and Toxicology Jamia Hamdard University Delhi India; ^2^ Amity Institute of Neuropsychology and Neurosciences Amity University Noida Uttar Pradesh India; ^3^ Department of Biosciences Jamia Millia Islamia Delhi India

**Keywords:** aging, Alzheimer’'s disease, circadian system, sleep wake cycle

## Abstract

Alzheimer's disease (AD) is considered to be the most typical form of dementia that provokes irreversible cognitive impairment. Along with cognitive impairment, circadian rhythm dysfunction is a fundamental factor in aggravating AD. A link among circadian rhythms, sleep, and AD has been well‐documented. The etiopathogenesis of circadian system disruptions and AD serves some general characteristics that also open up the possibility of viewing them as a mutually reliant path. In this review, we have focused on different factors that are related to circadian rhythm dysfunction. The various pathogenic factors, such as amyloid‐beta, neurofibrillary tangles, oxidative stress, neuroinflammation, and circadian rhythm dysfunction may all contribute to AD. In this review, we also tried to focus on melatonin which is produced from the pineal gland and can be used to treat circadian dysfunction in AD. Aside from amyloid beta, tau pathology may have a notable influence on sleep. Conclusively, the center of this review is primarily based on the principal mechanistic complexities associated with circadian rhythm disruption, sleep deprivation, and AD, and it also emphasizes the potential therapeutic strategies to treat and prevent the progression of AD.

## Graphical abstract

Amyloid beta plaques and accumulation of tangles are two major pathological hallmarks of Alzheimer's disease. Due to cholinergic disturbance, HPA axis dysfunction, neuronal loss, and retinal ganglion loss there is disturbance in circadian rhythm which leads to Alzheimer's disease dysfunction.

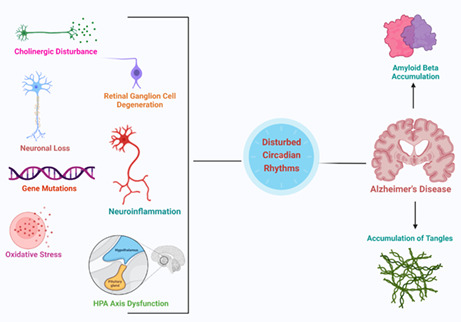

## 1 INTRODUCTION

Alzheimer's disease (AD) is the most common type of neurodegenerative disorder, which largely causes dementia and mainly affects older aged people. By the year 2050, around 12 million cases will be reported.^1^, ^2^ In AD, accumulation of amyloid beta and hyperphosphorylated tau are microscopic pathologies, whereas reduction in hippocampal volume, frontotemporal, and associated cortical atrophy with ventricular enlargement are macroscopic findings.^3^, ^4^, ^5^ To rule out AD, multiple biomarkers are available, like cerebrospinal fluid (CSF) molecules (for example, amyloid and tau), and to see atrophy in the brain, various neuroimaging techniques, such as computed tomography, magnetic resonance imaging, or positron emission tomography (PET). Current pharmacological treatments include donepezil, galantamine, and rivastigmine, which work as cholinesterase inhibitors. Memantine works as an N‐methyl D‐aspartate antagonist and Abun approved this in 2021.^6^, ^7^ Most current studies focus on the molecular aspect of AD, which mainly focuses on neuroinflammation, mitochondrial dysfunction, and glial cell activation.^8^ Currently, researchers focus on circadian rhythms, which help the researchers to understand AD pathophysiology in a relatively comprehensive and satisfactory way and also help to address or develop therapeutic targets of AD. Sleep disruptions and circadian disorders are quite common; around 45% of patients face problems with sleep.^9^, ^10^ These symptoms are present for several patients with AD even before the final medical diagnosis of AD. Based on multiple studies, it is seen that sleep disturbances can lead to neurodegeneration and even cognitive impairment. In the future, it can be utilized as a biomarker for neurodegeneration. In one study, it is seen that older women with diminished and irregular circadian rhythms have a higher risk of developing one of the types of impairments of AD, such as mild cognitive impairment and dementia. Various studies suggest that 25%–66% of patients with AD face sleep disruption, which can be easily noticeable.^11^, ^12^, ^13^, ^14^, ^15^, ^16^, ^17^ Melatonin (N‐acetyl 5–methoxytryptamine) is a hormone regulated by the circadian rhythms, and it plays a vital role in the neurodegenerative event of AD.^18^ The primary source of melatonin is the brain's pineal gland, but other organs like the retina, bone marrow, kidney, pancreas, skin, and glial cells are also involved. Melatonin is a multifunctional hormone that regulates circadian rhythm and shows anti‐inflammatory, cytoprotective, and anti‐oxidant properties. The circadian clock regulates melatonin and during a study in rat and mice models, melatonin shows the highest plasma melatonin level at midnight.^19^, ^20^ Melatonin production decreases with aging which can be considered a critical factor for the onset of AD. When impairment or disruption is seen in the suprachiasmatic nucleus (SCN), melatonin levels are reduced, resulting in circadian rhythm disruption.^21^, ^22^, ^23^ Even reduction in CSF is linked with melatonin, and, finally, melatonin progresses AD by causing oxidative damage in the AD brain. Patients with AD have a low level of melatonin as compared with healthy patients. Melatonin can be a promising therapeutic approach to inhibit AD progression as it has free radical scavenging properties as well as anti‐amyloidogenic properties. Melatonin also inhibits the secretion process of soluble amyloid precursor protein (APP) in various cell lines through APP maturation. Melatonin administration attenuates amyloid beta generation and deposition in vitro and in vivo models.^24^, ^25^, ^26^, ^27^, ^28^, ^29^, ^30^, ^31^, ^32^, ^33^, ^34^ A sundowning phenomenon enhances mental health decline, confusion, and agitation in patients with AD, whereas melatonin reduces the symptoms of sundowning and enhances cognition. In this review, we discuss the association of circadian dysfunction with AD pathology as well as a few pharmacological and non‐pharmacological interventions for sleep disruption in patients with AD.^35^, ^36^, ^37^, ^38^, ^39^

## 2 CIRCADIAN BIOLOGICAL CLOCK MECHANISM IN THE BRAIN

**FIGURE 1:**
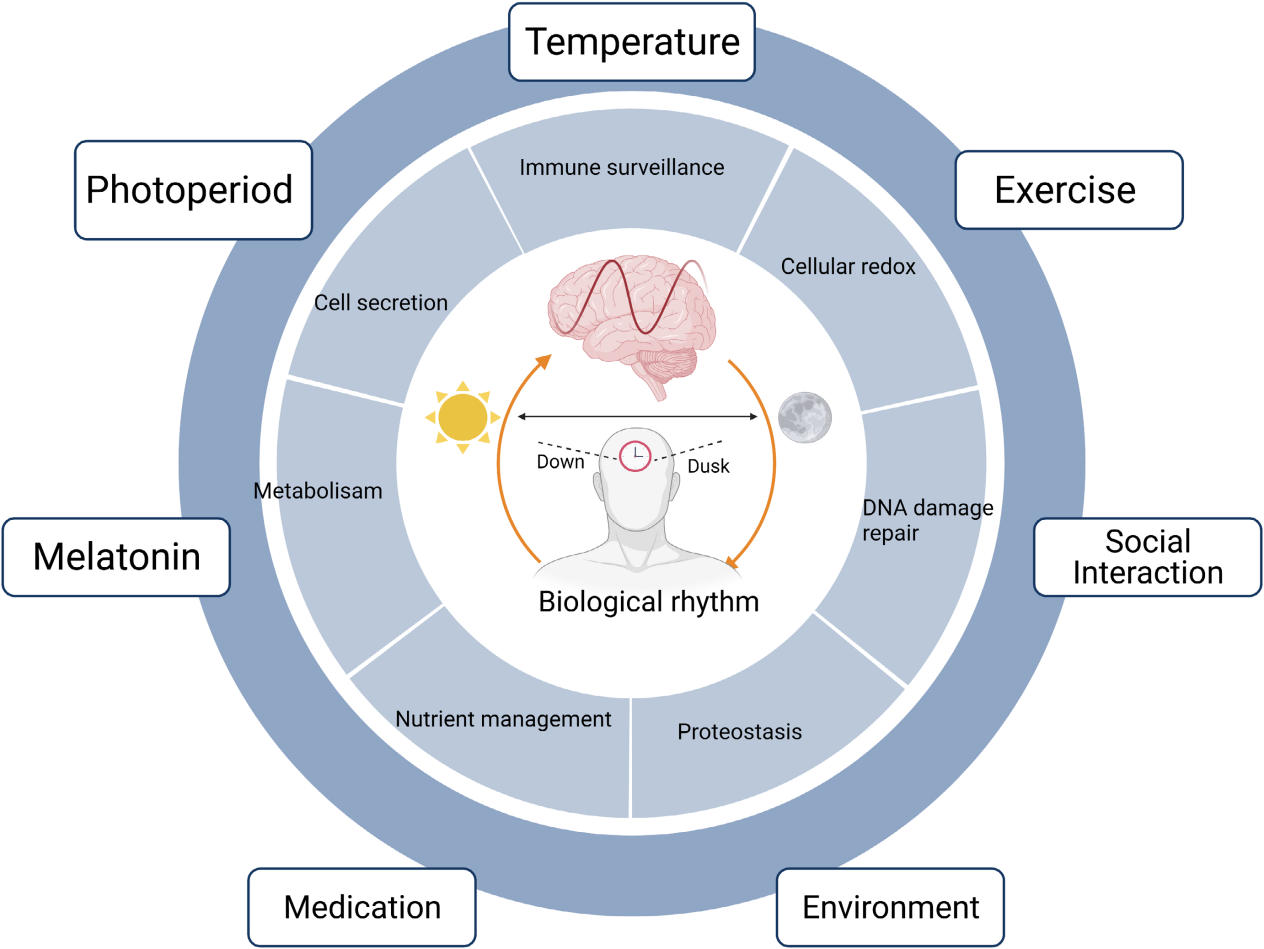
Twenty‐four hour biological clock in the human brain and its circadian disruption

**FIGURE 2:**
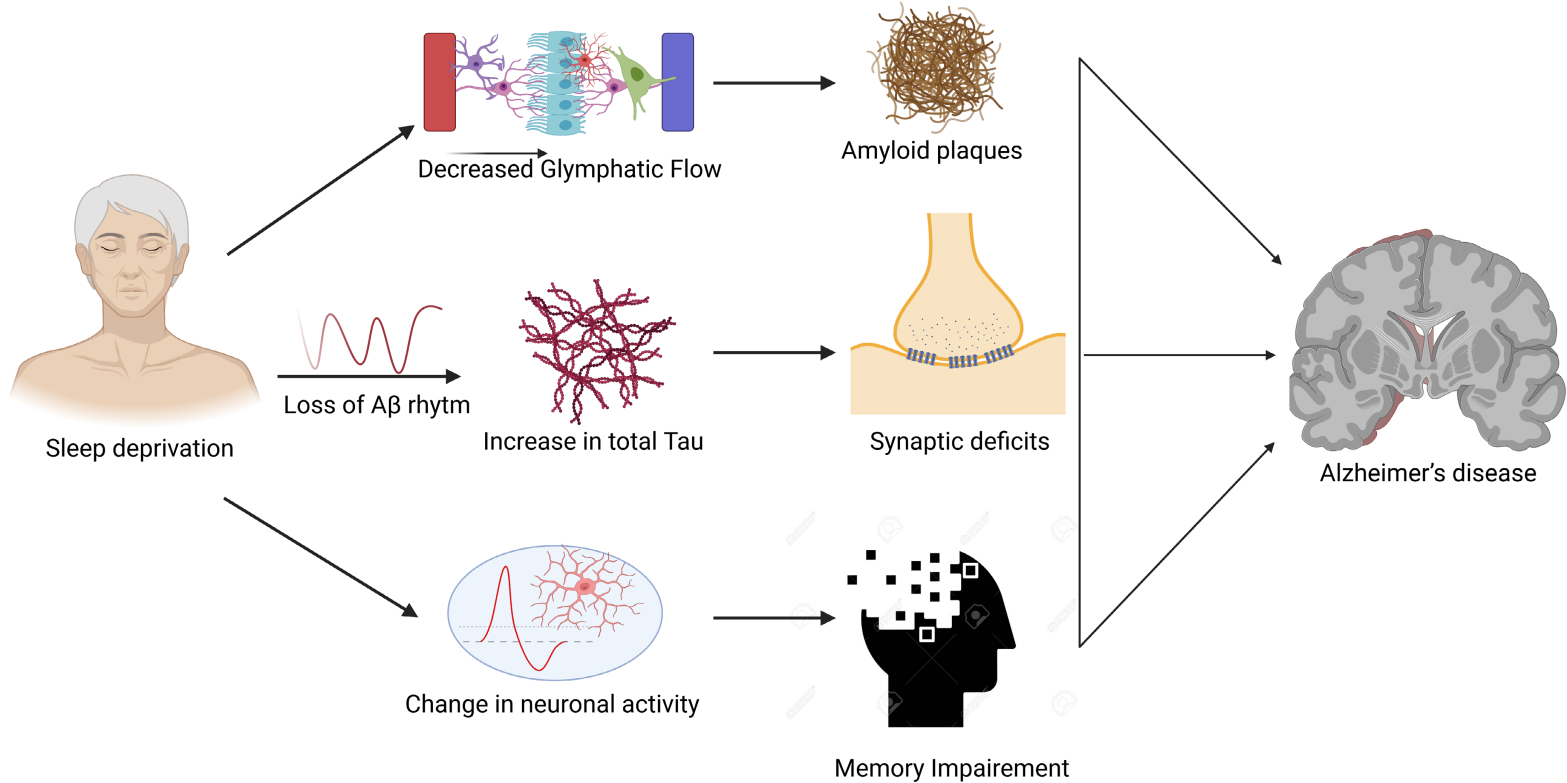
Crosstalk between sleep deprivation and Alzheimer's disease. Aβ, amyloid beta

**FIGURE 3:**
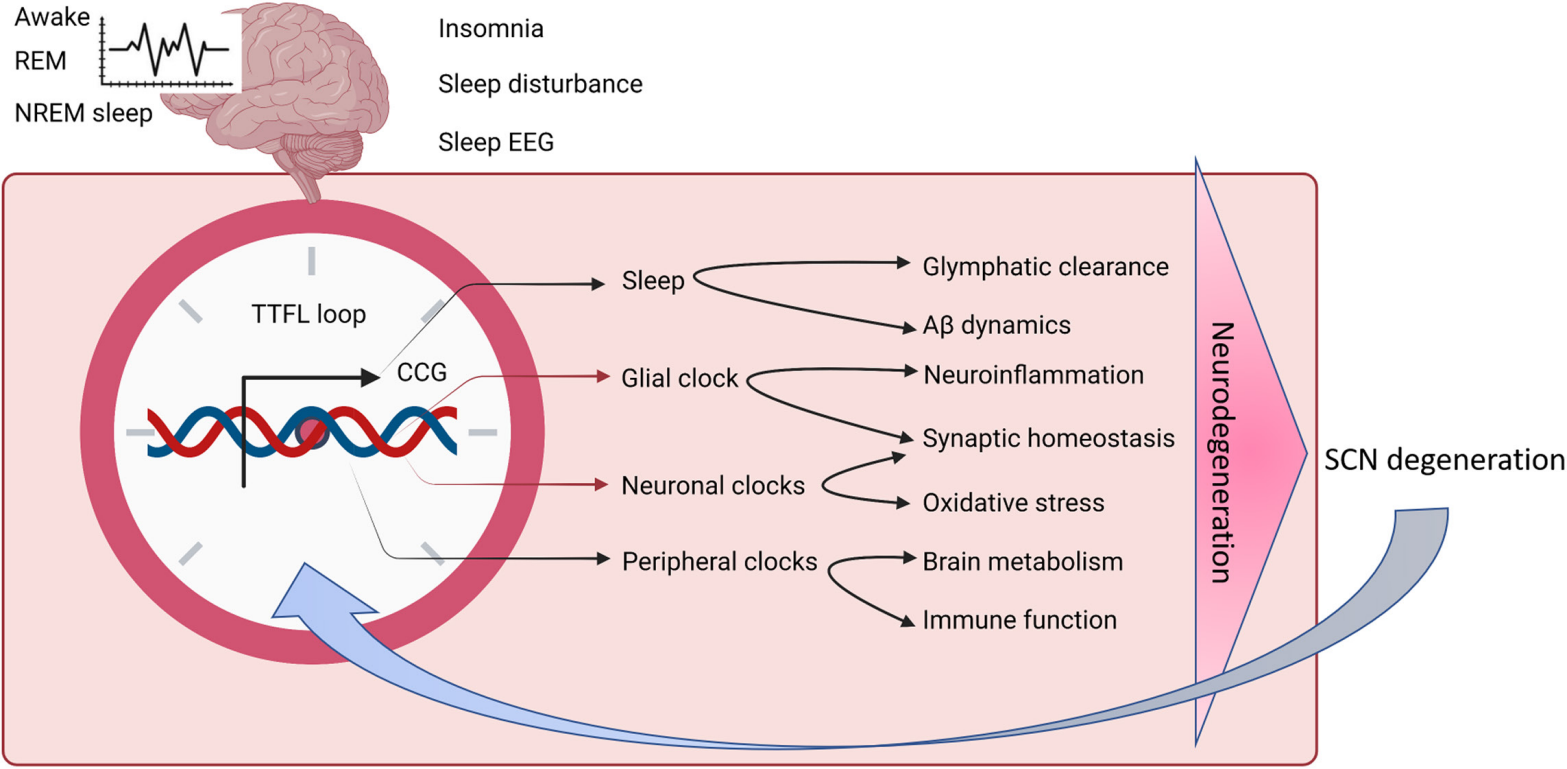
Linkage between circadian rhythm and Alzheimer's disease. Aβ, amyloid beta; EEG, electroencephalogram; nREM, non‐rapid eye movement; SCN, suprachiasmatic nucleus

A core gene of the circadian clock, the Period (*PER*) gene, was the first clock gene to be discovered by Jeffrey C. Hall and Michael Rosbach. The (PER protein is produced mainly at night and broken down during the day, and this whole cycle is regulated with the help of a negative feedback loop where PER protein blocks its production.^40^, ^41^ This protein is encoded by the *PER* gene. Recently, a new gene which is known as the double‐time (*DBT*) gene, has been discovered to encode DBT protein. The DBT protein averts the PER accumulation, proving that rhythm can be flagged according to the 24 hour biological clock. Circadian rhythm regulation is observed both at the central and peripheral levels. In 2017, Jeffrey C. Hall, Michael Rosbash, and Michael Wyong uncovered the molecular mechanisms regulating circadian rhythm and received the Nobel Prize in physiology or medicine. This mechanism demonstrates that mammals have a central pacemaker called the SCN in the hypothalamus. When the retina gets photic input, it transmits information to the SCN. This central clock regulates the circadian rhythm throughout all body functions through the peripheral autonomic nervous system and hormonal factors. The circadian system is a web of interlinked feedback loops and oscillators across all organisms. The Period (PER 1–3), Cryptochrome (CRY1 and 2), and Reverb (NR1D1 and NR1D2) genes are negative feedback regulators which suppress the positive limb. The SCN helps in the synchronization of cellular oscillators across organs in humans. The retina sends light and dark signals to the SCN, which further regulates it. It synchronizes the core clock oscillations in neurons, ultimately translated into oscillatory synaptic output, which transfers the signals to the multiple nuclei in the hypothalamus. All these patterns in neuronal activity, and behavioral and physiological arrhythmicity can be lost post ablation of the SCN.^40^, ^41^, ^42^, ^43^, ^44^, ^45^ The circadian clock system is shown in Figure 1, and relationship between circadian rhythm and AD is shown in Figures 2 and 3.

## 3 CHOLINERGIC DISTURBANCES AND CIRCADIAN DYSFUNCTION IN AD PATHOLOGY

Neurodegeneration can also be seen in the basal cholinergic forebrain. Disruption in circadian rhythm can also occur due to cells of the nucleus basalis magnocellularis, which projects to the SCN. Enrhardth reported that in rats, there are increased phase delays in response to lights when the cholinergic basal forebrain projects to the SCN. This study suggests a relationship between AD neurodegeneration and the circadian clock's signal entrainment ability.^46^, ^47^, ^48^

## 4 NEURONAL LOSS IN THE SCN AND CIRCADIAN DYSFUNCTION IN AD

During the autopsy of patients with AD, it was seen that there is a neuronal loss in the SCN, which is related to loss of amplitude in the circadian rest‐activity pattern. Apart from MT1, melatonin receptor expression was disturbed, which resulted in the SCN responding to the phase resetting signal and generating daily rhythms.^49^, ^50^

## 5 RETINAL GANGOLIAN CELL LOSS AND CIRCADIAN DYSFUNCTION IN AD

A particular type of subset of retinal ganglion cells (RGCs) known as Melanopsin expressing RGCs (mRGCs) was discovered in 2002. These cells are photoreceptors inside the retina, which help in the photoentrainment of circadian rhythms by projecting light to the SCN. Melanopsin expressing mRGCs constitutes 1%–2% of all RGCs, but they can direct signals to the SCN through the retinal hypothalamic tract. In patients with AD, mRGC loss can be seen, which can cause amyloid beta deposition, and lead to impairment of the entire RGCs even though there is a deposition of amyloid beta in mRGCs. The Toronto study shows interesting results involving retinal amyloid beta deposition in patients with AD. These findings will help better understand the pathology of retinal amyloid beta deposition in patients with AD. Amyloid beta deposition in mRGCs can lead to instability in transmitting the circadian signal of light from the retina to the SCN.^51^, ^52^, ^53^, ^54^, ^55^

## 6 CIRCADIAN GENE DELETION AND CIRCADIAN DYSFUNCTION IN AD

Deletion mutations in the circadian clock gene cause neuronal injury. Core circadian clock disruption is directly linked to neurodegeneration in AD. *BMAL1* is considered to be one of the core genes of the master clock, and a study conducted in mice has shown the deletion of *BMAL1* in the hippocampus and cortex. In mice, we observe normal behavioral rhythms and normal sleep wake cycles assessed by wheel running actigraphy and electroencephalogram, respectively, in the presence of severe cortical astrogliosis, synaptic degeneration, and oxidative brain region damage in specific *BMAL1* knockout mice. These mice are closely related to transcription multiple redox defenses linked with circadian impairment. Low levels of *BMAL1* in the brain also lead to neurodegeneration caused by mitochondrial toxin B nitropropionic acid. The data suggest that decreased *BMAL* mediated transcriptional exacerbate neurodegeneration in AD. Clock‐gene regulation and better insight into the linkage of clock genes and neurodegeneration require further research and a deeper understanding to examine such regulations.^56^, ^57^, ^58^, ^59^ The effect of different clock genes on animal models is shown in Table 1.

**TABLE 1:** Effect of different clock genes on different animal models

Subject no.	Different models	Effect of clock genes on different circadian models	References
1.	APP‐PS1 mouse model	Casein kinase 1 isoforms ε and δ with inhibitor PF‐670462 reduce amyloid and plaque size as well reduce Aβ signal in the prefrontal cortex and hippocampus, which proves chronotherapy as a promising tool to improve behavior in mice	^103^
2.	Two‐month‐old female APPSwe/PS1dE9 mice	Female APPSwe/PS1dE9 mice show abnormal locomotor activity in which clock gene expression of clock genes Per 1, Per 2, Cry 1, and Cry 2 was increased during night time compared to day type in wild type control mice as Cry 1 and Cry2 expression was low in APPSwe /PS1dE9 mice. This study proves APPSwe /PS1dE9 mice as a most promising AD model to test therapeutic agents related to behavioral and circadian rhythm changes.	^104^
3.	Cultured fibroblasts and brain samples	*BMAL1* is a positive regulator of the circadian clock, and in cultured fibroblasts, DNA methylation regulates *BMAL1* rhythms which is linked to circadian alteration in AD	^105^
4.	Tg 4510 mice	In Tg4510 mice, it is seen that there is tauopathy in SCN and even disruption in PER2 and *BMAL1* in the hypothalamus of Tg4510 mice. This study proves that tauopathy can lead to normal circadian clock function disruption.	^106^
5.	AD brain	In this study, the glial fibrillary acid protein in human astrocytes is suppressed as there is an elevation in CLOCK and BMAL, which cause functional impairment by inhibition of aerobic glycolysis in AD	^107^
6.	5XFAD mouse model	Rev‐erbα, a circadian repressor, decreases amyloid plaque number and size in the 5XFAD AD mouse model. Even Rev‐erbα show a neuroinflammatory effect, which proves Rev‐erbα as a novel therapeutic target.	^108^
7.	APP/PS1dE9 mice	In APP/PS1dE9 mice, there is an alteration of rhythmic expression patterns of BACE 1 and ApoE in the hippocampus, which is activated by E4BP4 and *BMAL1*, respectively. So, finally, study suggests that hippocampal clock and circadian oscillation of AD risk gene are regulated by orexin signaling.	^109^

Abbreviations: Aβ, amyloid beta; AD, Alzheimer's disease; SCN, suprachiasmatic nucleus.

## 7 MICROGLIA, ASTROCYTE, AND CIRCADIAN DYSFUNCTION IN AD

Activation of microglia and astrocyte leads to neuroinflammation, which ultimately causes neurodegeneration. Astrocyte activation can be observed to model clock gene deletion in the in vitro model. Even the inflammatory response of microglia leads to variation in the functional circadian clock. Rev‐Erb alpha regulates pro‐inflammatory cytokine production in macrophages. Finally, inflammation shows the effect of the circadian clock as both Rev‐Erb alpha suppressing *BMAL1* levels in macrophages in response to lipopolysaccharides. Therefore, the *BMAL1* expression in the surrounding glia and neurons can be suppressed by cortex inflammation causing impairment of *BMAL1*‐associated genes, ultimately leading to neurodegeneration.^56^, ^60^

## 8 OXIDATIVE STRESS AND CIRCADIAN DYSFUNCTION IN AD PATHOLOGY

Numerous studies support the presence of augmented oxidative stress in AD. Less concentration of glutathione and catalase with higher consumption of oxygen (20%–30%) and a higher amount of polyunsaturated fatty acids make the brain a highly vulnerable target for lipid peroxidation.^61^, ^62^, ^63^ Lipids peroxidation interrupts cellular functions, followed by neuronal membrane destruction, and the production of highly reactive electrophilic aldehydes, including acrolein, malondialdehyde, and 4 hydroxy 2‐nomial (elevated in AD brains).^64^, ^65^, ^66^ Oxidative stress also damages nucleic acid and proteins. The role of oxidative stress etiology in AD pathogenesis is still unknown. In 1985, the activity of antioxidants, like superoxide dismutase and glutathione peroxidase with oxidative damage in the day‐night cycle in the rat cerebral cortex, whereas in humans, anti‐oxidants and circadian rhythmicity protect cells from oxidative damage.^67^, ^68^, ^69^, ^70^ The levels of glutathione reductase, glutathione peroxidase, superoxide dismutase, catalase, uric acid, and peroxiredoxin are high in the morning. In contrast, ascorbic melatonin and plasma level are high in the evening or night. This proves that oxidative stress leads to oxidative damage with the progression of AD, which is ultimately regulated by circadian dysregulation.^71^

## 9 ERK/MARK AND CIRCADIAN DYSFUNCTION IN AD

Cognitive impairment is the first symptom observed in AD. Impairment, such as memory, is enhanced by short‐term stress and impaired by long‐term stress, and the number of dendritic synapses decreases due to high cortisol levels during chronic stress.^72^ The pathway primarily revolves around memory consolidation, and the level of phosphor‐ERK CAMP, phosphor CREB, and activity of PKA and MEK are associated with a circadian rhythm. Moreover, the SCN regulates the hippocampus' Camp/PKA/ERK/CREB signaling pathway.^73^, ^74^, ^75^ The CREB/ERK/PKA/CAMP signaling pathway increases during rapid eye movement sleep. They are even ablating the *BMAL1* gene results in reduced Per1 and PERK levels. A study reported that ERK appears overactivated and memory is improved by pharmacological inhibition of ERK in an AD mouse model, whereas memory impairment is seen due to reduction of pCREB level downstream of the ERK pathway.^76^ ERK signaling pathway is disrupted in AD due to amyloid beta^1^, ^2^, ^3^, ^4^, ^5^, ^6^, ^7^, ^8^, ^9^, ^10^, ^11^, ^12^, ^13^, ^14^, ^15^, ^16^, ^17^, ^18^, ^19^, ^20^, ^21^, ^22^, ^23^, ^24^, ^25^, ^26^, ^27^, ^28^, ^29^, ^30^, ^31^, ^32^, ^33^, ^34^, ^35^, ^36^, ^37^, ^38^, ^39^, ^40^, ^41^, ^42^ bind injury. Finally, ERK/MAPK signaling pathway is a common pathway that causes stress as circadian rhythm even plays a role in memory consolidation.^77^

## 10 HPA AXIS AND CIRCADIAN DYSFUNCTION IN AD

HPA axis activation promotes AD pathogenesis. Even reducing cortisol levels by taking dexamethasone does not show positive results in patients with AD; instead of cortisol levels, few approaches to decrease and modulate HPA axis activity can be a promising avenue for treating AD. Even amyloid beta promotes HPA axis activity and increases corticosterone. The HPA axis is one of the common pathways by which SCRD and stress increase amyloid beta production, leading to AD.^78^

## 11 HIPPOCAMPAL VOLUME AND CIRCADIAN DYSFUNCTION IN AD

Reduced hippocampal volume was observed in AD and different neurodegenerative and psychiatric disorders. It is hypothesized that prolonged sleep restriction or sleep disruption can cause a decrease in hippocampal neuronal cell proliferation and neuronal cell survival. Few preliminary clinical trials and observational studies suggest that regular physical exercise, cognitive stimulation, and general medical conditions can reduce hippocampal volume or atrophy, reverse hippocampal atrophy, or even expand the hippocampal size.^79^, ^80^

## 12 GLYMPHATIC SYSTEM AND CIRCADIAN DYSFUNCTION IN AD

The glymphatic system was first described in 2012, which consists of intestinal fluid that regulates brain amyloid clearance by the perivascular space surrounding blood vessels. Glymphatic system dysfunction also plays a vital role in the severity of AD. To date, no clinically approved system has been developed to evaluate the functionality of the glymphatic system in humans. Recently, the glymphatic system has even played a role in glaucoma pathogenesis, characterized by progressive degeneration of RGCs and amyloid beta accumulation. This activity is higher during sleep and low during wakefulness. Even body posture during sleep, especially lateral body position, may increase the rat's glymphatic transport. Further studies need to be done to see the relation of the glymphatic system with patients with AD.^11^, ^81^, ^82^

## 13 PROTEOSTATIS AND CIRCADIAN DYSFUNCTION IN AD

Amyloid beta and tau are specific protein hallmarks seen in AD. Heat shock factor 1 is a type of factor in which deletion alters circulation clock oscillation. Proteasomal degeneration of proteins display oscillations in circadian patterns and expected circadian clock timing requires an understanding of the proteasome function. It is still unknown how the circadian clock controls rhythmic protein degradation in the brain.^83^

## 14 VASCULAR AND CIRCADIAN DYSFUNCTION IN AD

Microvascular change is considered an essential factor in the development of AD. Cerebral vascular perfusion is also under the control of the circadian system. According to PET scans and simple‐photon emission computed tomography, people with moderate cognitive impairment and an increased risk of developing AD exhibit hypometabolism and cerebral hypoperfusion. Antihypertensive treatment has also been shown to reduce the risk of AD. Brain microvascular changes are critical to AD development, both pathologically and clinically. The circadian system regulates cerebral vascular circulation as well.^84^, ^85^, ^86^ Conroy et al investigated the daily regularity of cerebral blood flow velocity (CBFV) across 30 hours of continuous awake time. The findings of this study suggested that human CBFV probably follows an endogenous circadian rhythm, which will be investigated further in the context of cerebrovascular/cardiovascular events and cognitive function deterioration.^87^, ^88^, ^89^ Laser‐Doppler flowmetry revealed similar results in rats. The cerebral blood flow has a diurnal periodicity independent of locomotor activity and blood pressure changes. The effect of the circadian rhythm on brain metabolism and perfusion should be carefully considered in future studies on the role of vascular function in AD etiopathogenesis.^90^, ^91^, ^92^

## 15 METABOLIC CHANGES AND CIRCADIAN DYSFUNCTION IN AD

Circadian/sleep disruption may be mediated by metabolic changes in neurodegenerative disorders, particularly AD. Insulin resistance has been linked to an increased risk of AD in clinical studies, and childhood obesity can also cause cognitive impairment later in life apart from diabetes. Apolipoprotein E (APOE) is a key regulator of lipid metabolism found primarily in brain astrocytes. The APOE 4 allele can cause mitochondrial dysfunction, leading to insulin resistance and metabolic defects as a major risk factor for AD.^93^, ^94^, ^95^, ^96^, ^97^, ^98^ A recent study suggests that peripheral metabolic dysfunction plays a role in the development of AD‐related neuropathology. The clock regulates the majority of metabolic activity, and the loss of circadian clocks has been linked to cellular and system‐wide metabolic deficits. Sleep deprivation significantly impacts metabolism, including an increase in insulin resistance markers. Based on these findings, it is enticing to believe that sleep disruption increases the risk of AD by disrupting metabolism.^99^, ^100^, ^101^, ^102^

## 16 MELATONIN AS A PROMISING THERAPEUTIC TARGET FOR AD

In AD, melatonin has shown multiple beneficial effects, like prevention of mitochondrial dysfunction, inhibition of amyloid beta toxicity, free radical scavenging, and even circadian dysregulation like sundowning and sleep disturbances.^110^ Melatonin even has blood–brain barrier crossing capacity, anti‐oxidant properties, as well as balanced amphiphilicity. Amyloid beta peptides are mainly produced with the help of amyloidogenic beta‐amyloid precursor protein (beta APP). Amyloid beta 42 is the most neurotoxic form of amyloid beta. This beta pleated sheet peptide ultimately forms an aggregation of senile plaques in the brain in the form of amyloid fibrils that disrupts synaptic communications leading to abnormal function of neurons and neuronal death. As melatonin has anti‐oxidant, neuroprotective, and anti‐amyloidogenic properties, it might help in decreasing amyloid beta formation. Melatonin has shown effects on both in vivo and in vitro models.^111^, ^112^, ^113^, ^114^, ^115^ Hyperphosphorylated tau plays a crucial role in dealing with memory and cognitive impairment in AD. Neurodegeneration happens due to tau hyperphosphorylation. This tau phosphorylation and protein kinase A (PKA) overactivation in the isopropanol‐induced rat brain can be attenuated by melatonin. This process is followed in the neuroblastoma SHSY5Y cell line and N2a induced by calyculin A, okadaic acid, and wortmannin. Melatonin shows neuroprotective effects in the degeneration of the hippocampus and enhances cognitive effects. These effects are displayed through regulating GSK3 and CDK5 activities in hippocampal neurons. Melatonin inhibits the expression level of caspase 3, prostate apoptosis response 4 (Par4), and Bcl2 associated BAX, reducing neuronal death.^116^, ^117^, ^118^, ^119^, ^120^, ^121^ Melatonin has an anti‐oxidant property that reduces oxidative stress. In an experimental study, it was observed that NF‐KB commenced IL‐6 in amyloid beta treated brain slices can be inhibited by melatonin in a concentration‐dependent fashion. Melatonin injection (ie, 5 mg/kg, 0.1 to 10 mg/kg, and 10 mg/kg) in the rat in which melatonin shows anti‐inflammatory effects and reduces neuroinflammation by increasing ATP production, stimulating GPX activities, and even enhances SOD activity.^122^ Therefore, this evidence shows the anti‐neuroinflammatory effects of melatonin on AD.

## 17 RELATION AMONG EXERCISE, CIRCADIAN RHYTHM, AND AD

Various animal models show exercise chronobiotic properties. It is difficult to identify whether exercise has chronobiotic properties in humans because it is quite hard to differentiate the range of effects shown by exercise from multiple other factors, like food, social influences, and light.^123^ Non‐photic stimuli, on the other hand, appear to be capable of synchronizing circadian rhythms in people who are blind who lack sensitivity to light, and this helps them entrain to routine schedules without utilizing exogenous melatonin. A recent study related to circadian rhythms and AD has shown that when a person exercises just before habitual sleep, it accelerates circadian rhythm and if it is performed during habitual sleep time, it delays circadian rhythms.^124^, ^125^, ^126^ Exercise also affects the hippocampus, which plays a role in affecting sleep quality. It has also been reported that people who do exercise regularly on a daily basis have better sleep quality as well as less daytime sleepiness when compared to people who are inactive and do not exercise. As a result, it is still possible that exercise has a greater impact on older adults who face difficulty in sleeping. Exercises also enhance the cognitive part and show neural plasticity which is effective in normal aging as well as a treatment for AD.^127^, ^128^, ^129^, ^130^, ^131^, ^132^ Sleep after exercise has a well‐known effect on cognitive performance. According to the recent study findings, physical activity plays a huge role in diminishing the effects of poor sleep quality on cognitive functioning in older adult women. As a result, more research is needed to understand the mechanisms underlying exercise, sleep, and cognitive function that are linked in older adults.^133^, ^134^, ^135^, ^136^, ^137^, ^138^

## 18 CURRENT THERAPIES AND FUTURE IMPLICATIONS

Unfortunately, at present, we have limited pharmacological and non‐pharmacological interventions to manage sleep disturbance in patients with AD. In AD, current behavioral practices include limited caffeine and alcohol intake, regular exercise, and maintaining regular bed and wake times with ample light exposure upon waking.^60^ Sufficient daytime light exposure is crucial for patients with AD, mainly for institutionalized patients. Consistent light exposure may bring changes in dysfunctional circadian rhythms in AD and reduce the “sundowning.” Patients with moderate‐to‐severe AD were included in the melatonin and trazodone trials, but only patients with mild‐to‐moderate AD were included in the ramelteon study. Melatonin is considered a part of various clinical manifestations and treatment strategies of AD.^139^, ^140^, ^141^ Actigraphy is used to measure all primary sleep outcomes. Despite the absence of severe side effects, we still have no evidence to suggest that melatonin and trazodone improve sleep quality. More comprehensive clinical trials are desperately needed in this area, particularly those focusing on sleep and cognitive or pathological outcomes in AD. Suvorexant is the first US Food and Drug Administration (FDA)‐approved orexin receptor antagonist which can show effects on amyloid deposition and cognitive end points in early‐stage or presymptomatic AD. Melatonin supplementation on a regular basis may help patients with mild cognitive impairment improve their cognitive performance slightly. However, there appears to be conflicting evidence in mice regarding the effectiveness of melatonin supplementation in reducing amyloid plaques and other AD correlates. Ramelteon has been approved for insomnia, whereas tasimelteon is for the treatment of non‐24 hour sleep–wake disorder in the blind. Until now, these two drugs have not been tested for AD but can be more effective than melatonin. Researchers are trying to develop a drug that can directly target the circadian clock, although they are still in the early stages of development. Small molecules that can alter circadian oscillations' amplitude, frequency, and period have been discovered through high throughput screening. RevErb is a small molecule agonist of the nuclear receptor that can improve metabolic function in mice by directly affecting circadian rhythms. Finally, the right targeting of the circadian clock could be a promising remedial option for treating AD.^33^, ^34^

## 19 CONCLUSION

The pathology of AD (amyloid and tau) has been linked to circadian dysfunctions, and sleep disruptions are very common in patients with Alzheimer's disease that play an important role in disease succession and pathology. Moreover, circadian rhythms communicate with nearly all systems and risk factors involved in the growth and progression of AD. Recognizing early signs of AD, such as changes in sleep patterns and rest‐activity rhythm anomalies, could be useful in identifying early biomarkers for interference to prevent the formation of amyloid‐beta, neurofibrillary tangles and the succession of neurodegeneration. In patients with advanced AD, bright light therapy combined with chronobiotics is effective in treating sundowning characteristics and other cognitive symptoms. Future research into the function of circadian misalignment in the initial stages of AD could lead to new preventive and therapeutic approaches. As a result, circadian rhythms are an excellent target for combating pathology.

## AUTHOR CONTRIBUTIONS

*Manuscript writing and drawing figures:* Faizan Ahmad. *Manuscript writing, reviewing, and editing:* Punya Sachdeva. *Editing:* Jasmine Sarkar. *Reviewing:* Rafiah Izhaar.

## FUNDING INFORMATION

No funding was received for this study.

## CONFLICT OF INTEREST

The authors declare they have no conflict of interest.

## References

[1] Sachdeva P , Ahmad F . In silico characterization of predominant genes involved in early onset Alzheimer's disease. J Neurobehav Sci. 2021;8:179‐190. doi:10.4103/jnbs.jnbs_34_21

[2] Huang Y , Mucke L . Alzheimer mechanisms and therapeutic strategies. Cell. 2012;148(6):1204‐1222. doi:10.1016/j.cell.2012.02.040 22424230 PMC3319071

[3] Masters CL , Simms G , Weinman NA , Multhaup G , McDonald BL , Beyreuther K . Amyloid plaque core protein in Alzheimer disease and down syndrome. Proc Natl Acad Sci U S A. 1985;82(12):4245‐4249. doi:10.1073/pnas.82.12.4245 3159021 PMC397973

[4] Glenner GG , Wong CW . Alzheimer's disease: initial report of the purification and characterization of a novel cerebrovascular amyloid protein. Biochem Biophys Res Commun. 1984;120(3):885‐890. doi:10.1016/s0006-291x(84)80190-4 6375662

[5] Hardy JA , Higgins GA . Alzheimer's disease: the amyloid cascade hypothesis. Science. 1992;256(5054):184‐185. doi:10.1126/science.1566067 1566067

[6] Hardy J . The discovery of Alzheimer‐causing mutations in the APP gene and the formulation of the "amyloid cascade hypothesis". FEBS J. 2017;284(7):1040‐1044. doi:10.1111/febs.14004 28054745

[7] Regen F , Hellmann‐Regen J , Costantini E , Reale M . Neuroinflammation and Alzheimer's disease: implications for microglial activation. Curr Alzheimer Res. 2017;14(11):1140‐1148. doi:10.2174/1567205014666170203141717 28164764

[8] Hong S , Dissing‐Olesen L , Stevens B . New insights on the role of microglia in synaptic pruning in health and disease. Curr Opin Neurobiol. 2016;36:128‐134. doi:10.1016/j.conb.2015.12.004 26745839 PMC5479435

[9] Holth J , Patel T , Holtzman DM . Sleep in Alzheimer's disease ‐ beyond amyloid. Neurobiol Sleep Circadian Rhythms. 2017;2:4‐14. doi:10.1016/j.nbscr.2016.08.002 28217760 PMC5312809

[10] Volicer L , Harper DG , Manning BC , Goldstein R , Satlin A . Sundowning and circadian rhythms in Alzheimer's disease. Am J Psychiatry. 2001;158(5):704‐711. doi:10.1176/appi.ajp.158.5.704 11329390

[11] Musiek ES , Holtzman DM . Mechanisms linking circadian clocks, sleep, and neurodegeneration. Science. 2016;354(6315):1004‐1008. doi:10.1126/science.aah4968 27885006 PMC5219881

[12] Mullard A . BACE inhibitor bust in Alzheimer trial. Nat rev Drug Discov. 2017;16(3):155. doi:10.1038/nrd.2017.43 28248932

[13] Mullard A . Alzheimer amyloid hypothesis lives on. Nat rev Drug Discov. 2016;16(1):3‐5. doi:10.1038/nrd.2016.281 28031570

[14] Huang CW , Lui CC , Chang WN , Lu CH , Wang YL , Chang CC . Elevated basal cortisol level predicts lower hippocampal volume and cognitive decline in Alzheimer's disease. J Clin Neurosci. 2009;16(10):1283‐1286. doi:10.1016/j.jocn.2008.12.026 19570680

[15] Selkoe DJ , Hardy J . The amyloid hypothesis of Alzheimer's disease at 25 years. EMBO mol Med. 2016;8(6):595‐608. doi:10.15252/emmm.201606210 27025652 PMC4888851

[16] Lleó A , Greenberg SM , Growdon JH . Current pharmacotherapy for Alzheimer's disease. Annu rev Med. 2006;57:513‐533. doi:10.1146/annurev.med.57.121304.131442 16409164

[17] Sala Frigerio C , De Strooper B . Alzheimer's disease mechanisms and emerging roads to novel therapeutics. Annu rev Neurosci. 2016;39:57‐79. doi:10.1146/annurev-neuro-070815-014015 27050320

[18] Srinivasan V , Pandi‐Perumal SR , Cardinali DP , Poeggeler B , Hardeland R . Melatonin in Alzheimer's disease and other neurodegenerative disorders. Behav Brain Funct. 2006;2:15. doi:10.1186/1744-9081-2-15 16674804 PMC1483829

[19] Matsubara E , Bryant‐Thomas T , Pacheco Quinto J , et al. Melatonin increases survival and inhibits oxidative and amyloid pathology in a transgenic model of Alzheimer's disease published correction appears. J Neurochem. 2003;86(5):1312. J Neurochem. 2003;85(5):1101–1108. doi:10.1046/j.1471-4159.2003.01654.x 12753069

[20] Feng Z , Chang Y , Cheng Y , et al. Melatonin alleviates behavioral deficits associated with apoptosis and cholinergic system dysfunction in the APP 695 transgenic mouse model of Alzheimer's disease. J Pineal Res. 2004;37(2):129‐136. doi:10.1111/j.1600-079X.2004.00144.x 15298672

[21] Poeggeler B , Miravalle L , Zagorski MG , et al. Melatonin reverses the profibrillogenic activity of apolipoprotein E4 on the Alzheimer amyloid Abeta peptide. Biochemistry. 2001;40(49):14995‐15001. doi:10.1021/bi0114269 11732920

[22] Quinn J , Kulhanek D , Nowlin J , et al. Chronic melatonin therapy fails to alter amyloid burden or oxidative damage in old Tg2576 mice: implications for clinical trials. Brain Res. 2005;1037(1–2):209‐213. doi:10.1016/j.brainres.2005.01.023 15777772

[23] Hardeland R . Cognitive enhancers in moderate to severe Alzheimer's disease. Clin Med Insights: Ther. 2011. doi:10.4137/CMT.S6344

[24] Adlard PA , Cherny RA , Finkelstein DI , et al. Rapid restoration of cognition in Alzheimer's transgenic mice with 8‐hydroxy quinoline analogs is associated with decreased interstitial Abeta. Neuron. 2008;59(1):43‐55. doi:10.1016/j.neuron.2008.06.018 18614028

[25] Crouch PJ , Savva MS , Hung LW , et al. The Alzheimer's therapeutic PBT2 promotes amyloid‐β degradation and GSK3 phosphorylation via a metal chaperone activity. J Neurochem. 2011;119(1):220‐230. doi:10.1111/j.1471-4159.2011.07402.x 21797865

[26] Brusco LI , Fainstein I , Márquez M , Cardinali DP . Effect of melatonin in selected populations of sleep‐disturbed patients. Biol Signals Recept. 1999;8(1–2):126‐131. doi:10.1159/000014580 10085474

[27] Mishima K , Okawa M , Hozumi S , Hishikawa Y . Supplementary administration of artificial bright light and melatonin as potent treatment for disorganized circadian rest‐activity and dysfunctional autonomic and neuroendocrine systems in institutionalized demented elderly persons. Chronobiol Int. 2000;17(3):419‐432. doi:10.1081/cbi-100101055 10841214

[28] Cohen‐Mansfield J , Garfinkel D , Lipson S . Melatonin for treatment of sundowning in elderly persons with dementia ‐ a preliminary study. Arch Gerontol Geriatr. 2000;31(1):65‐76. doi:10.1016/s0167-4943(00)00068-6 10989165

[29] Furio AM , Brusco LI , Cardinali DP . Possible therapeutic value of melatonin in mild cognitive impairment: a retrospective study. J Pineal Res. 2007;43(4):404‐409. doi:10.1111/j.1600-079X.2007.00491.x 17910609

[30] Cardinali DP , Brusco LI , Liberczuk C , Furio AM . The use of melatonin in Alzheimer's disease. Neuro Endocrinol Lett. 2002;23(Suppl 1):20‐23. 12019347

[31] Asayama K , Yamadera H , Ito T , Suzuki H , Kudo Y , Endo S . Double blind study of melatonin effects on the sleep‐wake rhythm, cognitive and non‐cognitive functions in Alzheimer type dementia. J Nippon Med Sch. 2003;70(4):334‐341. doi:10.1272/jnms.70.334 12928714

[32] Mahlberg R , Kunz D , Sutej I , Kühl KP , Hellweg R . Melatonin treatment of day‐night rhythm disturbances and sundowning in Alzheimer disease: an open‐label pilot study using actigraphy. J Clin Psychopharmacol. 2004;24(4):456‐459. doi:10.1097/01.jcp.0000132443.12607.fd 15232344

[33] Dowling GA , Burr RL , Van Someren EJ , et al. Melatonin and bright‐light treatment for rest‐activity disruption in institutionalized patients with Alzheimer's disease. J Am Geriatr Soc. 2008;56(2):239‐246. doi:10.1111/j.1532-5415.2007.01543.x 18070004 PMC2642966

[34] Singer C , Tractenberg RE , Kaye J , et al. A multicenter, placebo‐controlled trial of melatonin for sleep disturbance in Alzheimer's disease. Sleep. 2003;26(7):893‐901. doi:10.1093/sleep/26.7.893 14655926 PMC4418658

[35] Farajnia S , Deboer T , Rohling JH , Meijer JH , Michel S . Aging of the suprachiasmatic clock. Neuroscientist. 2014;20(1):44‐55. doi:10.1177/1073858413498936 23924666

[36] Hofman MA , Swaab DF . Living by the clock: the circadian pacemaker in older people. Ageing Res rev. 2006;5(1):33‐51. doi:10.1016/j.arr.2005.07.001 16126012

[37] Hofman MA , Swaab DF . Alterations in circadian rhythmicity of the vasopressin‐producing neurons of the human suprachiasmatic nucleus (SCN) with aging. Brain Res. 1994;651(1–2):134‐142. doi:10.1016/0006-8993(94)90689-0 7922560

[38] Swaab DF , Fliers E , Partiman TS . The suprachiasmatic nucleus of the human brain in relation to sex, age and senile dementia. Brain Res. 1985;342(1):37‐44. doi:10.1016/0006-8993(85)91350-2 4041816

[39] Hofman MA . Lifespan changes in the human hypothalamus. Exp Gerontol. 1997;32(4–5):559‐575. doi:10.1016/s0531-5565(96)00162-3 9315457

[40] Rosbash M . A 50‐year personal journey: location, gene expression, and circadian rhythms. Cold Spring Harb Perspect Biol. 2017;9(12):a032516. doi:10.1101/cshperspect.a032516 28600396 PMC5710103

[41] Nobelprize.org . Scientific background: discoveries of molecular mechanisms controlling the circadian rhythm; 2017.

[42] Sahab UM , Al Mamun A . Circadian rhythms: biological clock of living organisms. Biol Med. 2018;10:1. A+. Gale OneFile: Health and Medicine. https://link.gale.com/apps/doc/A531467556/HRCA?u=anon~773246a0&sid=googleScholar&xid=d7d0ae15.Accessed July 14, 2022

[43] Pett JP , Korenčič A , Wesener F , Kramer A , Herzel H . Feedback loops of the mammalian circadian clock constitute Repressilator. PLoS Comput Biol. 2016;12(12):e1005266. doi:10.1371/journal.pcbi.1005266 27942033 PMC5189953

[44] Vosshall LB , Price JL , Sehgal A , Saez L , Young MW . Block in nuclear localization of period protein by a second clock mutation, timeless. Science. 1994;263(5153):1606‐1609. doi:10.1126/science.812824 8128247

[45] Bargiello TA , Jackson FR , Young MW . Restoration of circadian behavioural rhythms by gene transfer in drosophila. Nature. 1984;312(5996):752‐754. doi:10.1038/312752a0 6440029

[46] Price JL , Blau J , Rothenfluh A , Abodeely M , Kloss B , Young MW . Double‐time is a novel drosophila clock gene that regulates PERIOD protein accumulation. Cell. 1998;94(1):83‐95. doi:10.1016/s0092-8674(00)81224-6 9674430

[47] Colwell CS , Kaufman CM , Menaker M . Phase‐shifting mechanisms in the mammalian circadian system: new light on the carbachol paradox. J Neurosci. 1993;13(4):1454‐1459. doi:10.1523/JNEUROSCI.13-04-01454.1993 7681871 PMC6576708

[48] Coogan AN , Schutová B , Husung S , et al. The circadian system in Alzheimer's disease: disturbances, mechanisms, and opportunities. Biol Psychiatry. 2013;74(5):333‐339. doi:10.1016/j.biopsych.2012.11.021 23273723

[49] Mattis J , Sehgal A . Circadian rhythms, sleep, and disorders of aging. Trends Endocrinol Metab. 2016;27(4):192‐203. doi:10.1016/j.tem.2016.02.003 26947521 PMC4808513

[50] Slats D , Claassen JA , Verbeek MM , Overeem S . Reciprocal interactions between sleep, circadian rhythms and Alzheimer's disease: focus on the role of hypocretin and melatonin. Ageing Res Rev. 2013;12(1):188‐200. doi:10.1016/j.arr.2012.04.003 22575905

[51] Erhardt C , Galani R , Jeltsch H , et al. Modulation of photic resetting in rats by lesions of projections to the suprachiasmatic nuclei expressing p75 neurotrophin receptor. Eur J Neurosci. 2004;19(7):1773‐1788. doi:10.1111/j.1460-9568.2004.03281.x 15078551

[52] Berson DM , Dunn FA , Takao M . Phototransduction by retinal ganglion cells that set the circadian clock. Science. 2002;295(5557):1070‐1073. doi:10.1126/science.1067262 11834835

[53] Koronyo Y , Biggs D , Barron E , et al. Retinal amyloid pathology and proof‐of‐concept imaging trial in Alzheimer's disease. JCI Insight. 2017;2(16):e93621. doi:10.1172/jci.insight.93621 28814675 PMC5621887

[54] Feng R , Li L , Yu H , Liu M , Zhao W . Melanopsin retinal ganglion cell loss and circadian dysfunction in Alzheimer's disease (review). Mol Med Rep. 2016;13(4):3397‐3400. doi:10.3892/mmr.2016.4966 26935586 PMC4805057

[55] La Morgia C , Ross‐Cisneros FN , Koronyo Y , et al. Melanopsin retinal ganglion cell loss in Alzheimer disease. Ann Neurol. 2016;79(1):90‐109. doi:10.1002/ana.24548 26505992 PMC4737313

[56] Musiek ES , Lim MM , Yang G , et al. Circadian clock proteins regulate neuronal redox homeostasis and neurodegeneration. J Clin Invest. 2013;123(12):5389‐5400. doi:10.1172/JCI70317 24270424 PMC3859381

[57] Levy‐Lahad E , Wasco W , Poorkaj P , et al. Candidate gene for the chromosome 1 familial Alzheimer's disease locus. Science. 1995;269(5226):973‐977. doi:10.1126/science.7638622 7638622

[58] Bélanger V , Picard N , Cermakian N . The circadian regulation of Presenilin‐2 gene expression. Chronobiol Int. 2006;23(4):747‐766. doi:10.1080/07420520600827087 16887746

[59] Song H , Moon M , Choe HK , et al. Aβ‐induced degradation of BMAL1 and CBP leads to circadian rhythm disruption in Alzheimer's disease. Mol Neurodegener. 2015;10:13. doi:10.1186/s13024-015-0007-x 25888034 PMC4404698

[60] Musiek ES , Xiong DD , Holtzman DM . Sleep, circadian rhythms, and the pathogenesis of Alzheimer disease. Exp Mol Med. 2015;47(3):e148. doi:10.1038/emm.2014.121 25766617 PMC4351409

[61] Cermakian N , Lange T , Golombek D , et al. Crosstalk between the circadian clock circuitry and the immune system. Chronobiol Int. 2013;30(7):870‐888. doi:10.3109/07420528.2013.782315 23697902 PMC7195843

[62] Kalsbeek A , van der Spek R , Lei J , Endert E , Buijs RM , Fliers E . Circadian rhythms in the hypothalamo‐pituitary‐adrenal (HPA) axis. Mol Cell Endocrinol. 2012;349(1):20‐29. doi:10.1016/j.mce.2011.06.042 21782883

[63] Zaplatic E , Bule M , Shah SZA , Uddin MS , Niaz K . Corrigendum toMolecular mechanisms underlying protective role of quercetin in attenuating Alzheimer's disease" [Life Sci. 221 (2019) 109–119]. Life Sci. 2019;231:116616. doi:10.1016/j.lfs.2019.116616 31326083

[64] Suzuki K . Regulation of inflammatory responses by the autonomic nervous system. Nihon Rinsho Meneki Gakkai Kaishi. 2016;39(2):96‐102. doi:10.2177/jsci.39.96 27212595

[65] Scheiermann C , Kunisaki Y , Frenette PS . Circadian control of the immune system. Nat Rev Immunol. 2013;13(3):190‐198. doi:10.1038/nri3386 23391992 PMC4090048

[66] Moreira PI , Carvalho C , Zhu X , Smith MA , Perry G . Mitochondrial dysfunction is a trigger of Alzheimer's disease pathophysiology. Biochim Biophys Acta. 2010;1802(1):2‐10. doi:10.1016/j.bbadis.2009.10.006 19853658

[67] Beal MF . Oxidatively modified proteins in aging and disease. Free Radic Biol Med. 2002;32(9):797‐803. doi:10.1016/s0891-5849(02)00780-3 11978481

[68] Dang TN , Arseneault M , Murthy V , Ramassamy C . Potential role of acrolein in neurodegeneration and in Alzheimer's disease. Curr Mol Pharmacol. 2010;3(2):66‐78. 20302565

[69] Singh M , Dang TN , Arseneault M , Ramassamy C . Role of by‐products of lipid oxidation in Alzheimer's disease brain: a focus on acrolein. J Alzheimers Dis. 2010;21(3):741‐756. doi:10.3233/JAD-2010-100405 20634576

[70] Bradley‐Whitman MA , Lovell MA . Biomarkers of lipid peroxidation in Alzheimer disease (AD): an update. Arch Toxicol. 2015;89(7):1035‐1044. doi:10.1007/s00204-015-1517-6 25895140 PMC4466146

[71] Breteler MM . Vascular risk factors for Alzheimer's disease: an epidemiologic perspective. Neurobiol Aging. 2000;21(2):153‐160. doi:10.1016/s0197-4580(99)00110-4 10867200

[72] Schwabe L , Joëls M , Roozendaal B , Wolf OT , Oitzl MS . Stress effects on memory: an update and integration. Neurosci Biobehav rev. 2012;36(7):1740‐1749. doi:10.1016/j.neubiorev.2011.07.002 21771612

[73] Finsterwald C , Alberini CM . Stress and glucocorticoid receptor‐dependent mechanisms in long‐term memory: from adaptive responses to psychopathologies. Neurobiol Learn Mem. 2014;112:17‐29. doi:10.1016/j.nlm.2013.09.017 24113652 PMC3979509

[74] Eckel‐Mahan KL , Phan T , Han S , et al. Circadian oscillation of hippocampal MAPK activity and cAmp: implications for memory persistence. Nat Neurosci. 2008;11(9):1074‐1082. doi:10.1038/nn.2174 19160506 PMC2772165

[75] Phan TX , Chan GC , Sindreu CB , Eckel‐Mahan KL , Storm DR . The diurnal oscillation of MAP (mitogen‐activated protein) kinase and adenylyl cyclase activities in the hippocampus depends on the suprachiasmatic nucleus [published correction appears in J Neurosci. 2011 Aug 10;31(32):11744. Phan, Trongha H [corrected to Phan, Trongha X]]. J Neurosci. 2011;31(29):10640‐10647. doi:10.1523/JNEUROSCI.6535-10.2011 21775607 PMC3146036

[76] Masters CL , Bateman R , Blennow K , Rowe CC , Sperling RA , Cummings JL . Alzheimer's disease. Nat Rev Dis Primers. 2015;1:15056. Published 2015 Oct 15. doi:10.1038/nrdp.2015.56 27188934

[77] Faucher P , Mons N , Micheau J , Louis C , Beracochea DJ . Hippocampal injections of oligomeric amyloid β‐peptide (1‐42) induce selective working memory deficits and long‐lasting alterations of ERK signaling pathway. Front Aging Neurosci. 2016;7:245. doi:10.3389/fnagi.2015.00245 26793098 PMC4707555

[78] Park HJ , Ran Y , Jung JI , et al. The stress response neuropeptide CRF increases amyloid‐β production by regulating γ‐secretase activity. EMBO J. 2015;34(12):1674‐1686. doi:10.15252/embj.201488795 25964433 PMC4475401

[79] Brureau A , Zussy C , Delair B , et al. Deregulation of hypothalamic‐pituitary‐adrenal axis functions in an Alzheimer's disease rat model. Neurobiol Aging. 2013;34(5):1426‐1439. doi:10.1016/j.neurobiolaging.2012.11.015 23273603

[80] Kent BA , Mistlberger RE . Sleep and hippocampal neurogenesis: implications for Alzheimer's disease. Front Neuroendocrinol. 2017;45:35‐52. doi:10.1016/j.yfrne.2017.02.004 28249715

[81] Yan T , Qiu Y , Yu X , Yang L . Glymphatic dysfunction: a bridge between sleep disturbance and mood disorders. Front Psych. 2021;12:658340. doi:10.3389/fpsyt.2021.658340 PMC8138157 34025481

[82] Reeves BC , Karimy JK , Kundishora AJ , et al. Glymphatic system impairment in Alzheimer's disease and idiopathic Normal pressure hydrocephalus. Trends Mol Med. 2020;26(3):285‐295. doi:10.1016/j.molmed.2019.11.008 31959516 PMC7489754

[83] Hastings MH , Goedert M . Circadian clocks and neurodegenerative diseases: time to aggregate? Curr Opin Neurobiol. 2013;23(5):880‐887. doi:10.1016/j.conb.2013.05.004 23797088 PMC3782660

[84] Borenstein AR , Wu Y , Mortimer JA , et al. Developmental and vascular risk factors for Alzheimer's disease. Neurobiol Aging. 2005;26(3):325334. doi:10.1016/j.neurobiolaging.2004.04.010 15639310

[85] Berti V , Pupi A , Mosconi L . PET/CT in diagnosis of dementia. Ann N Y Acad Sci. 2011;1228:81‐92. doi:10.1111/j.1749-6632.2011.06015.x 21718326 PMC3692287

[86] Habert MO , Horn JF , Sarazin M , et al. Brain perfusion SPECT with an automated quantitative tool can identify prodromal Alzheimer's disease among patients with mild cognitive impairment. Neurobiol Aging. 2011;32(1):15‐23. doi:10.1016/j.neurobiolaging.2009.01.013 19250707

[87] Johnson KA , Jones K , Holman BL , et al. Preclinical prediction of Alzheimer's disease using SPECT. Neurology. 1998;50(6):1563‐1571. doi:10.1212/wnl.50.6.1563 9633695

[88] de la Torre JC . Vascular basis of Alzheimer's pathogenesis. Ann N Y Acad Sci. 2002;977:196‐215. doi:10.1111/j.1749-6632.2002.tb04817.x 12480752

[89] Forette F , Seux ML , Staessen JA , et al. Prevention of dementia in randomised double‐blind placebo‐controlled systolic hypertension in Europe (syst‐Eur) trial. Lancet. 1998;352(9137):1347‐1351. doi:10.1016/s0140-6736(98)03086-4 9802273

[90] Yasar S , Xia J , Yao W , et al. Antihypertensive drugs decrease risk of Alzheimer disease: ginkgo evaluation of memory study. Neurology. 2013;81(10):896‐903. doi:10.1212/WNL.0b013e3182a35228 23911756 PMC3885216

[91] Conroy DA , Spielman AJ , Scott RQ . Daily rhythm of cerebral blood flow velocity. J Circadian Rhythms. 2005;3(1):3. doi:10.1186/1740-3391-3-3 15760472 PMC555580

[92] Wauschkuhn CA , Witte K , Gorbey S , Lemmer B , Schilling L . Circadian periodicity of cerebral blood flow revealed by laser‐doppler flowmetry in awake rats: relation to blood pressure and activity. Am J Physiol Heart Circ Physiol. 2005;289(4):H1662‐H1668. doi:10.1152/ajpheart.01242.2004 15894567

[93] Willette AA , Bendlin BB , Starks EJ , et al. Association of Insulin Resistance with Cerebral Glucose Uptake in late middle‐aged adults at risk for Alzheimer disease [published correction appears in JAMA Neurol. 2015 Dec;72(12):1537] [published correction appears in JAMA Neurol. 2017 Jul 1;74(7):873]. JAMA Neurol. 2015;72(9):1013‐1020. doi:10.1001/jamaneurol.2015.0613 26214150 PMC4570876

[94] Luciano R , Barraco GM , Muraca M , et al. Biomarkers of Alzheimer disease, insulin resistance, and obesity in childhood. Pediatrics. 2015;135(6):1074‐1081. doi:10.1542/peds.2014-2391 25963004

[95] Huang Y , Mahley RW . Apolipoprotein E: structure and function in lipid metabolism, neurobiology, and Alzheimer's diseases. Neurobiol Dis. 2014;72(Pt A):3‐12. doi:10.1016/j.nbd.2014.08.025 25173806 PMC4253862

[96] Szendroedi J , Phielix E , Roden M . The role of mitochondria in insulin resistance and type 2 diabetes mellitus. Nat Rev Endocrinol. 2011;8(2):92‐103. doi:10.1038/nrendo.2011.138 21912398

[97] Lane‐Donovan C , Philips GT , Herz J . More than cholesterol transporters: lipoprotein receptors in CNS function and neurodegeneration. Neuron. 2014;83(4):771‐787. doi:10.1016/j.neuron.2014.08.005 25144875 PMC4240629

[98] Dibner C , Schibler U . Circadian timing of metabolism in animal models and humans. J Intern Med. 2015;277(5):513‐527. doi:10.1111/joim.12347 25599827

[99] Maury E , Hong HK , Bass J . Circadian disruption in the pathogenesis of metabolic syndrome. Diabetes Metab. 2014;40(5):338‐346. doi:10.1016/j.diabet.2013.12.005 24433933

[100] Weljie AM , Meerlo P , Goel N , et al. Oxalic acid and diacylglycerol 36:3 are cross‐species markers of sleep debt. Proc Natl Acad Sci USA. 2015;112(8):2569‐2574. doi:10.1073/pnas.1417432112 25675494 PMC4345602

[101] Davies SK , Ang JE , Revell VL , et al. Effect of sleep deprivation on the human metabolome. Proc Natl Acad Sci USA. 2014;111(29):10761‐10766. doi:10.1073/pnas.1402663111 25002497 PMC4115565

[102] Taheri S , Lin L , Austin D , Young T , Mignot E . Short sleep duration is associated with reduced leptin, elevated ghrelin, and increased body mass index. PLoS Med. 2004;1(3):e62. doi:10.1371/journal.pmed.0010062 15602591 PMC535701

[103] Sundaram S , Nagaraj S , Mahoney H , et al. Inhibition of casein kinase 1δ/εimproves cognitive‐affective behavior and reduces amyloid load in the APP‐PS1 mouse model of Alzheimer's disease published correction appears. Sci Rep. 2019;9(1):15167. doi:10.1038/s41598-019-50197-x 31619739 PMC6795822

[104] Oyegbami O , Collins HM , Pardon MC , Ebling FJP , Heery DM , Moran PM . Abnormal clock gene expression and locomotor activity rhythms in two month‐old female APPSwe/PS1dE9 mice. Curr Alzheimer Res. 2017;14(8):850‐860. doi:10.2174/1567205014666170317113159 28317486

[105] Cronin P , McCarthy MJ , Lim ASP , et al. Circadian alterations during early stages of Alzheimer's disease are associated with aberrant cycles of DNA methylation in BMAL1. Alzheimers Dement. 2017;13(6):689‐700. doi:10.1016/j.jalz.2016.10.003 27883893 PMC5785929

[106] Stevanovic K , Yunus A , Joly‐Amado A , et al. Disruption of normal circadian clock function in a mouse model of tauopathy. Exp Neurol. 2017;294:58‐67. doi:10.1016/j.expneurol.2017.04.015 28461004

[107] Yoo ID , Park MW , Cha HW , et al. Elevated CLOCK and BMAL1 contribute to the impairment of aerobic glycolysis from astrocytes in Alzheimer's disease. Int J Mol Sci. 2020;21(21):7862. doi:10.3390/ijms21217862 33114015 PMC7660350

[108] Lee J , Kim DE , Griffin P , et al. Inhibition of REV‐ERBs stimulates microglial amyloid‐beta clearance and reduces amyloid plaque deposition in the 5XFAD mouse model of Alzheimer's disease. Aging Cell. 2020;19(2):e13078. doi:10.1111/acel.13078 31800167 PMC6996949

[109] Ma Z , Jiang W , Zhang EE . Orexin signaling regulates both the hippocampal clock and the circadian oscillation of Alzheimer's disease‐risk genes. Sci Rep. 2016;6:36035. doi:10.1038/srep36035 27796320 PMC5086843

[110] Hossain MF , Uddin MS , Uddin GMS , et al. Melatonin in Alzheimer's disease: a latent endogenous regulator of neurogenesis to mitigate Alzheimer's neuropathology. Mol Neurobiol. 2019;56(12):8255‐8276. doi:10.1007/s12035-019-01660-3 31209782

[111] Gastel JA , Roseboom PH , Rinaldi PA , Weller JL , Klein DC . Melatonin production: proteasomal proteolysis in serotonin N‐acetyltransferase regulation. Science. 1998;279(5355):1358‐1360. doi:10.1126/science.279.5355.1358 9478897

[112] Carrillo‐Vico A , Lardone PJ , Alvarez‐Sánchez N , Rodríguez‐Rodríguez A , Guerrero JM . Melatonin: buffering the immune system. Int J Mol Sci. 2013;14(4):8638‐8683. doi:10.3390/ijms14048638 23609496 PMC3645767

[113] Hardeland R , Cardinali DP , Srinivasan V , Spence DW , Brown GM , Pandi‐Perumal SR . Melatonin‐‐a pleiotropic, orchestrating regulator molecule. Prog Neurobiol. 2011;93(3):350‐384. doi:10.1016/j.pneurobio.2010.12.004 21193011

[114] Okatani Y , Wakatsuki A , Kaneda C . Melatonin increases activities of glutathione peroxidase and superoxide dismutase in fetal rat brain. J Pineal Res. 2000;28(2):89‐96. doi:10.1034/j.1600-079x.2001.280204.x 10709970

[115] Hardeland R . Antioxidative protection by melatonin: multiplicity of mechanisms from radical detoxification to radical avoidance. Endocrine. 2005;27(2):119‐130. doi:10.1385/endo:27:2:119 16217125

[116] Kang JW , Hong JM , Lee SM . Melatonin enhances mitophagy and mitochondrial biogenesis in rats with carbon tetrachloride‐induced liver fibrosis. J Pineal Res. 2016;60(4):383‐393. doi:10.1111/jpi.12319 26882442

[117] Leon J , Acuña‐Castroviejo D , Sainz RM , Mayo JC , Tan DX , Reiter RJ . Melatonin and mitochondrial function. Life Sci. 2004;75(7):765‐790. doi:10.1016/j.lfs.2004.03.003 15183071

[118] Song J , Whitcomb DJ , Kim BC . The role of melatonin in the onset and progression of type 3 diabetes published correction appears. Mol Brain. 2017;10(1):59. Mol Brain. 2017;10(1):35. doi:10.1186/s13041-017-0333-8 28764741 PMC5539639

[119] Wu YH , Feenstra MG , Zhou JN , et al. Molecular changes underlying reduced pineal melatonin levels in Alzheimer disease: alterations in preclinical and clinical stages. J Clin Endocrinol Metab. 2003;88(12):5898‐5906. doi:10.1210/jc.2003-030833 14671188

[120] Lin L , Huang QX , Yang SS , Chu J , Wang JZ , Tian Q . Melatonin in Alzheimer's disease. Int J Mol Sci. 2013;14(7):14575‐14593. Published 2013 Jul 12. doi:10.3390/ijms140714575 23857055 PMC3742260

[121] Urrestarazu E , Iriarte J . Clinical management of sleep disturbances in Alzheimer's disease: current and emerging strategies. Nat Sci Sleep. 2016;8:21‐33. doi:10.2147/NSS.S76706 26834500 PMC4716729

[122] Laste G , de Macedo IC , Ripoll Rozisky J , Ribeiro da Silva F , Caumo W , Torres IL . Melatonin administration reduces inflammatory pain in rats. J Pain Res. 2012;5:359‐362. doi:10.2147/JPR.S34019 23204863 PMC3508662

[123] Mrosovsky N . Locomotor activity and non‐photic influences on circadian clocks. Biol Rev Camb Philos Soc. 1996;71(3):343‐372. doi:10.1111/j.1469-185x.1996.tb01278.x 8761159

[124] Hastings MH , Duffield GE , Smith EJ , Maywood ES , Ebling FJ . Entrainment of the circadian system of mammals by nonphotic cues. Chronobiol Int. 1998;15(5):425‐445. doi:10.3109/07420529808998700 9787934

[125] Mistlberger RE , Skene DJ . Nonphotic entrainment in humans? J Biol Rhythms. 2005;20(4):339‐352. doi:10.1177/0748730405277982 16077153

[126] Youngstedt SD . Effects of exercise on sleep. Clin Sports Med. 2005;24(2):355‐365. doi:10.1016/j.csm.2004.12.003 15892929

[127] Driver HS , Taylor SR . Exercise and sleep. Sleep Med Rev. 2000;4(4):387‐402. doi:10.1053/smrv.2000.0110 12531177

[128] Youngstedt SD , O'Connor PJ , Dishman RK . The effects of acute exercise on sleep: a quantitative synthesis. Sleep. 1997;20(3):203‐214. doi:10.1093/sleep/20.3.203 9178916

[129] Huber R , Ghilardi MF , Massimini M , Tononi G . Local sleep and learning. Nature. 2004;430(6995):78‐81. doi:10.1038/nature02663 15184907

[130] Colcombe S , Kramer AF . Fitness effects on the cognitive function of older adults: a meta‐analytic study. Psychol Sci. 2003;14(2):125‐130. doi:10.1111/1467-9280.t01-1-01430 12661673

[131] Colcombe SJ , Kramer AF , Erickson KI , et al. Cardiovascular fitness, cortical plasticity, and aging. Proc Natl Acad Sci USA. 2004;101(9):3316‐3321. doi:10.1073/pnas.0400266101 14978288 PMC373255

[132] Liu‐Ambrose T , Nagamatsu LS , Graf P , Beattie BL , Ashe MC , Handy TC . Resistance training and executive functions: a 12‐month randomized controlled trial. Arch Intern Med. 2010;170(2):170‐178. doi:10.1001/archinternmed.2009.494 20101012 PMC3448565

[133] Liu‐Ambrose T , Nagamatsu LS , Voss MW , Khan KM , Handy TC . Resistance training and functional plasticity of the aging brain: a 12‐month randomized controlled trial. Neurobiol Aging. 2012;33(8):1690‐1698. doi:10.1016/j.neurobiolaging.2011.05.010 21741129

[134] Nagamatsu LS , Chan A , Davis JC , et al. Physical activity improves verbal and spatial memory in older adults with probable mild cognitive impairment: a 6‐month randomized controlled trial. J Aging Res. 2013;2013:861893. doi:10.1155/2013/861893 23509628 PMC3595715

[135] Nagamatsu LS , Handy TC , Hsu CL , Voss M , Liu‐Ambrose T . Resistance training promotes cognitive and functional brain plasticity in seniors with probable mild cognitive impairment. Arch Intern Med. 2012;172(8):666‐668. Erratum in: Arch Intern Med. 2013 Aug 12;173(15):1477. doi:10.1001/archinternmed.2012.379 22529236 PMC3514552

[136] Lautenschlager NT , Cox KL , Flicker L , et al. Effect of physical activity on cognitive function in older adults at risk for Alzheimer disease: a randomized trial. JAMA. 2008;300(9):1027‐1037. Erratum in: JAMA. 2009 Jan 21;301(3):276. doi:10.1001/jama.300.9.1027 18768414

[137] Baker LD , Frank LL , Foster‐Schubert K , et al. Effects of aerobic exercise on mild cognitive impairment: a controlled trial. Arch Neurol. 2010;67(1):71‐79. doi:10.1001/archneurol.2009.307 20065132 PMC3056436

[138] Lambiase MJ , Gabriel KP , Kuller LH , Matthews KA . Sleep and executive function in older women: the moderating effect of physical activity. J Gerontol A Biol Sci Med Sci. 2014;69(9):1170‐1176. doi:10.1093/gerona/glu038 24744391 PMC4441058

[139] Ancoli‐Israel S , Klauber MR , Jones DW , et al. Variations in circadian rhythms of activity, sleep, and light exposure related to dementia in nursing‐home patients. Sleep. 1997;20(1):18‐23. PMID: 9130329. 9130329

[140] Shochat T , Martin J , Marler M , Ancoli‐Israel S . Illumination levels in nursing home patients: effects on sleep and activity rhythms. J Sleep Res. 2000;9(4):373‐379. doi:10.1046/j.1365-2869.2000.00221.x 11386204

[141] McCleery J , Cohen DA , Sharpley AL . Pharmacotherapies for sleep disturbances in Alzheimer's disease. Cochrane Database Syst Rev. 2014;(3):CD009178. doi:10.1002/14651858.CD009178.pub2 24659320

